# Atypical body movements during night in young children with autism spectrum disorder: a pilot study

**DOI:** 10.1038/s41598-019-43397-y

**Published:** 2019-05-06

**Authors:** Nobushige Naito, Mitsuru Kikuchi, Yuko Yoshimura, Hirokazu Kumazaki, Sachiko Kitagawa, Takashi Ikeda, Chiaki Hasegawa, Daisuke N. Saito, Sarah Tomiyama, Yoshio Minabe

**Affiliations:** 1 Department of Psychiatry & Behavioral Science, Graduate School of Medical Science, Kanazawa University, Kanazawa, 920-8640 Japan; 2 Research Center for Child Mental Development, Kanazawa University, Kanazawa, 920-8640 Japan; 3 Institute of Human and Social Sciences, Kanazawa University, Kanazawa, 920-1192 Japan

**Keywords:** Diagnostic markers, Autism spectrum disorders

## Abstract

Children with autism spectrum disorder (ASD) reportedly suffer from sleep problems at a higher rate than typically developing (TD) children. Several previous studies have reported differences in sleep indices (e.g., sleep latency) in children with ASD. However, no previous studies have focused specifically on the time course of body movements. In the present study, we investigated the time course of body movements in young TD children and young children with ASD as well as the relationship between body movements during night and social ability. Seventeen TD children and 17 children with ASD participated in this study (5 to 8 years old). We used an accelerometer attached to the waist to record movements during night and measured the average time course of body movements for 3 nights. Our results demonstrated that the rate of body movement 2 to 3 hours after the onset of body stillness was higher in children with ASD than in TD children. In addition, the higher rate of body movement at 0.5 to 1 hour after the onset of body stillness was associated with a lower social ability in the children with ASD. Our results suggested that the time course of body movements is an objective behavioural index for young children with ASD.

## Introduction

Autism spectrum disorder (ASD) comprises a set of neurodevelopmental disorders characterized by deficits in social communication and restrictive and repetitive patterns of behaviour, interests and activities^1^. Numerous previous studies have indicated that children with ASD experience more sleep problems than typically developing (TD) children^2–4^.

Polysomnography (PSG) is considered to be the gold standard for assessing the physiology and disturbances of sleep; it can document long motionless periods of wakefulness, sleep apnoea and periodic movements, which are difficult to document using simpler methods such as actigraphy. Using PSG, some previous studies have compared sleep between children with ASD and non-autistic children^5,6^ and demonstrated lower sleep efficiency, decreased total sleep time or shorter REM latency in children with ASD^7–11^. To highlight the need for additional evidence demonstrating the relationship between social ability and the quality of sleep, only two reports involving young children with ASD have been published using objective methods (i.e., PSG). One previous study demonstrated that severe ASD symptoms were associated with a shorter total sleep time and wakefulness after sleep onset^8^, and the other study demonstrated that severe ASD symptoms were associated with less slow-wave sleep^11^.

While carers are considered good informants regarding children’s sleep behaviour, actigraphy (a movement-based index measured by an accelerometer) is recommended as an objective technique for home monitoring of the timing, duration, and continuity of sleep^12^. Especially when investigating young children, the use of PSG in an unfamiliar environment (i.e., the novel environment of a sleep laboratory) could be stressful and might not be appropriate for accurately evaluating daily sleep. Children with ASD may also have more difficulty tolerating PSG due to tactile sensitivities. Therefore, to objectively measure daily sleep, the use of an accelerometer, which can be carried out in the usual environment at home, is a very practical method. Previous studies using actigraphy in school-aged^13,14^ and preschool-aged children with ASD have been reported^10,15–19^, and abnormalities in the actigraphy parameters (i.e., longer sleep latency, lower sleep efficiency and shorter total sleep time) have been reported in children with ASD. However, no reports involving young children with ASD have been published on the relationship between social ability and the quality of sleep measured by actigraphy.

**Figure 1:**
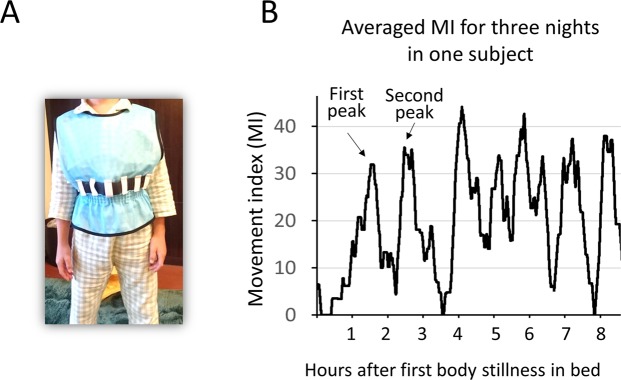
Accelerometer and time-series data of the movement index (MI) after the first onset of body stillness. (**A**) We used a wristwatch-like accelerometer attached to the waist and continuously recorded movement during night. (**B**) Time-series data of the movement index (MI) after the first onset of body stillness in a TD child. Averaged time-series data of the movement index (MI) over three nights were used in the analysis. Periodic increases in body movements that likely corresponded to the REM sleep periods were observed in each subject.

In previous actigraphy studies using validated algorithms, time-series data were scored as sleep or wake, and ultimately, software-estimated sleep parameters were determined, such as total sleep time, sleep efficiency, wake after sleep onset, and sleep latency. All previous sleep studies using actigraphy in children with ASD have reported abnormalities using these estimated sleep parameters. Intriguingly, with the convenience of accelerometers, fine motor skills in infants at heightened vs. low risk for ASD^20^ and locomotor dynamics in children with ASD^21^ have recently been reported. However, no previous studies have focused on the precise time course of body movements after the onset of body stillness in the bed in young children with ASD (e.g., at what time point do they move more after the onset of body stillness in the bed, which can be evaluated using raw time-series data) using accelerometers or its relationship with symptom severity. In the present study, our main purpose was to investigate the precise time course of body movements after the onset of body stillness in the bed in young children with ASD. As described, using PSG, one recent study reported that less deep sleep is associated with the higher severity of symptoms in children with ASD^11^. Given that there is frequent body movement during light sleep and wake conditions^22^, nocturnal frequent body movement is considered to reflect less deep sleep. Therefore, we hypothesized that children with ASD would be characterized by an atypical time course of body movement at night, and this time course would be related to the severity of symptoms in children with ASD. In the present study, we used a wristwatch-like accelerometer attached to the waist (Fig. 1A) to continuously record movements during night.

## Results

### Differences in the movement index (MI) between TD children and children with ASD

The demographic data of all subjects are presented in Table 1.

**Table 1:** Demographic characteristics.

	TD	ASD	*P*-value
Number of participants	17	17	
Gender (male/female)	11/6	13/4	n.s.
Age in months, mean (range)	71.1 (61–79)	77.1 (60–98)	n.s.
School/preschool-age	7/10	10/7	n.s.
Usual sleep time reported by carers (SD)	9.45 (0.54) hours	9.51 (0.59) hours	n.s.
Usual sleep quality reported by carers (SD)*	5.0 (0.71)	5.0 (0.87)	n.s.
K-ABC Mental Processing Scale (SD)	102.8 (10.5)	93.9 (18.9)	n.s.
SRS total score (SD)	46.0 (5.73)	69.4 (10.46)	<0.0001
ADOS social communication total score (SD)	—	10 (3.58)	
ADOS restricted and repetitive behaviours score (SD)	—	1.53 (0.94)	
Vineland-II, MBS (SD)	15.4 (1.8)	19.5 (2.9)	<0.0001
ADHD-RS, total score (SD)	3.9 (4.1)	18.7 (8.7)	<0.0001

*Carers subjectively rated their child’s sleep quality using the following six-point rating scale: 1 = very bad, 2 = bad, 3 = somewhat bad, 4 = somewhat good, 5 = good, and 6 = very good. K-ABC, Kaufman Assessment Battery for Children. SRS, Social Responsiveness Scale. ADOS, Autism Diagnostic Observation Schedule. Vineland-II, Vineland Adaptive Behavior Scales second edition. MBS, Maladaptive Behavior Scale. ADHD-RS, attention-deficit hyperactivity disorder rating scale. The values indicate the mean values (range or standard deviation). n.s., not significant.

**Figure 2:**
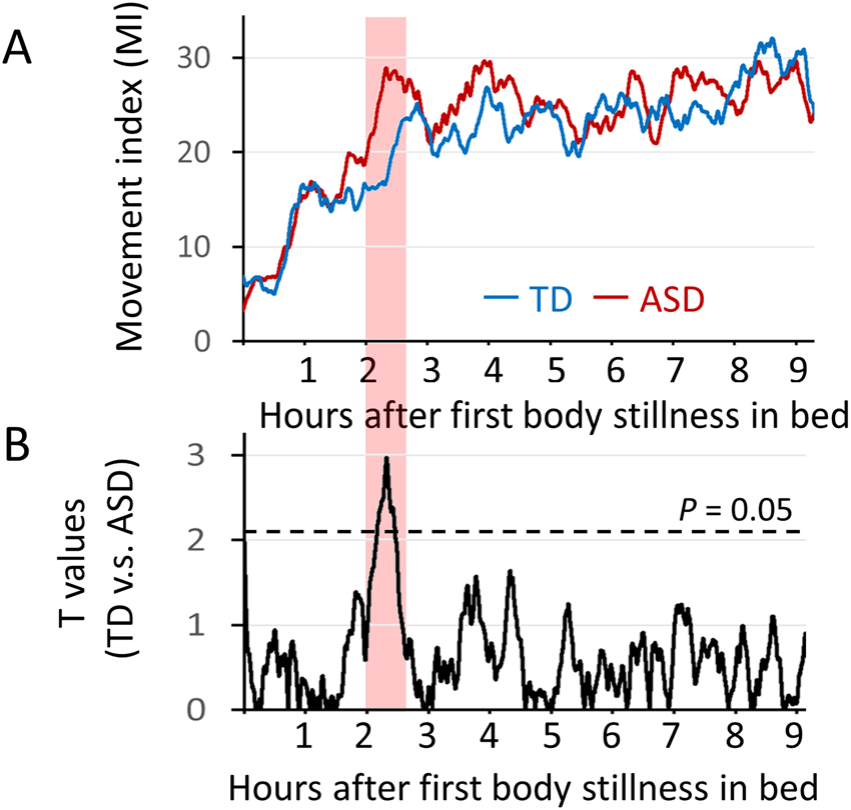
Overall averaged time-series data of the movement index (MI) after the first onset of body stillness in TD children (n = 17) and children with ASD (n = 17). (**A**) The blue line shows the time series of the MI in the TD children, and the red line shows the time series of the MI in the children with ASD. (**B**) Time-series data of the MI in the TD children and children with ASD were compared using unpaired two-tailed t-tests for each time window (20 minutes). Broken lines show the threshold of P = 0.05 (t = 2.04).

According to the unpaired two-tailed *t*-tests performed to analyse each time window during the 9-hour period after the first onset of body stillness in the bed, significant differences existed during the time window of 2 to 3 hours after the first onset of body stillness (Fig. 2B). The MI was significantly higher in the children with ASD than in the TD children during this time period.

In addition, we added complementary analyses using data for one day, two days (average), and three days (average) to compare TD children and children with ASD. As the number of days increased, the significant difference after 2–3 hours between TD children and children with ASD became clearer, and the other spiky T value waveform decreased (Fig. S1).

### Correlations between the movement index (MI) and social ability

**Figure 3:**
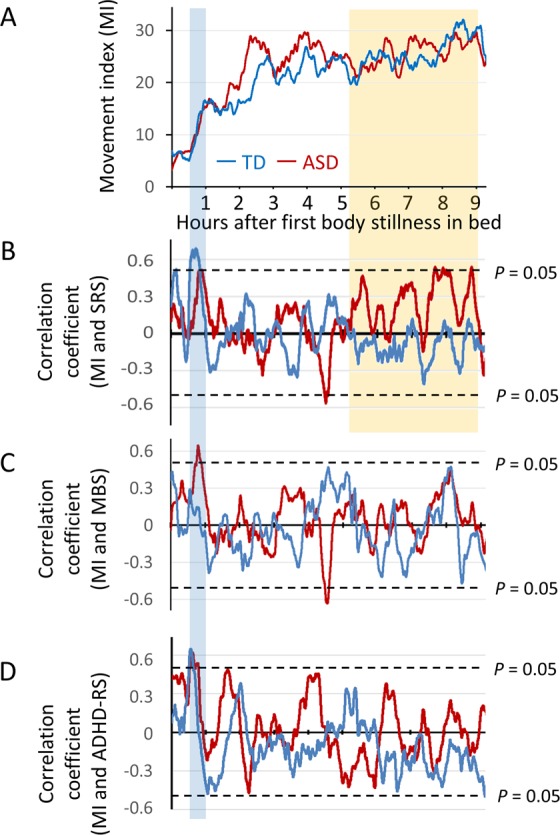
Correlations between the movement index (MI) and behavioural indices in each time window. (**A**) Overall averaged time-series data of the MI after the first onset of body stillness in the TD children (n = 17) and children with ASD (n = 17). (**B**) Time series of the correlation coefficients between the MI and social ability as evaluated by the SRS total scores. A positive value indicated that a higher rate of body movement was associated with a lower social ability. (**C**) Time series of the correlation coefficients between the MI and adaptive behaviour as evaluated by the Vineland Adaptive Behavior Scales. A positive value indicated that a higher rate of body movement was associated with severe maladaptive behaviour. (**D**) Time series of the correlation coefficients between the MI and ADHD symptoms as evaluated by the ADHD-RS. A positive value indicated that a higher rate of body movement was associated with higher ADHD symptoms.

We employed the social responsiveness scale (SRS)^23,24^, which is distinct from other measures in that it provides a continuous measure of social ability (from impaired to above average) instead of a categorical yes/no identification of ASD impairments. High scores are associated with more severe social impairments. The SRS has been widely adopted in genetic research on ASD because it can measure social ability in all family members (those with ASD and those without). Significant positive correlations were observed between the MI during the time window of 0.5 to 1 hour after the first onset of body stillness and the total score on the SRS in both groups. This positive relationship indicated that a higher rate of body movement was associated with a lower social ability. In addition, significant positive correlations between the MI and SRS scores were observed during the latter half of night (i.e., from 5 to 9 hours after the first onset of body stillness) in the children with ASD (Fig. 3B).

### Correlations between the movement index (MI) and adaptive behaviour

Significant positive correlations were observed between the MI and the Maladaptive Behavior Scale (MBS) of the Vineland Adaptive Behavior Scales second edition (Vineland-II)^25^ during the time window of 0.5 to 1 hour after the first onset of body stillness in the children with ASD (blue shaded area; Fig. 3C). This positive relationship indicated that a higher rate of body movement was associated with frequent maladaptive behaviour.

### Correlations between the movement index (MI) and ADHD symptoms

Significant positive correlations were observed between the MI and the attention-deficit hyperactivity disorder (ADHD) symptoms of the ADHD rating scales^26^ during the time window of 0.5 to 1 hour after the first onset of body stillness in the TD children and the children with ASD (blue shaded area; Fig. 3D). This positive relationship indicated that a higher rate of body movement was associated with ADHD symptoms.

### Differences in the latency of the peaks of the movement index (MI) between TD children and children with ASD

There were no significant differences in the peaks of the MI. The latency to the first peak was 1.09 ± 0.26 hours (mean ± standard deviation) in the TD children and 1.02 ± 0.28 hours in the children with ASD. The latency to the second peak was 2.39 ± 0.39 hours in the TD children and 2.28 ± 0.43 hours in the children with ASD.

### Correlations between the latency to the peak of the movement index (MI) and social ability

**Figure 4:**
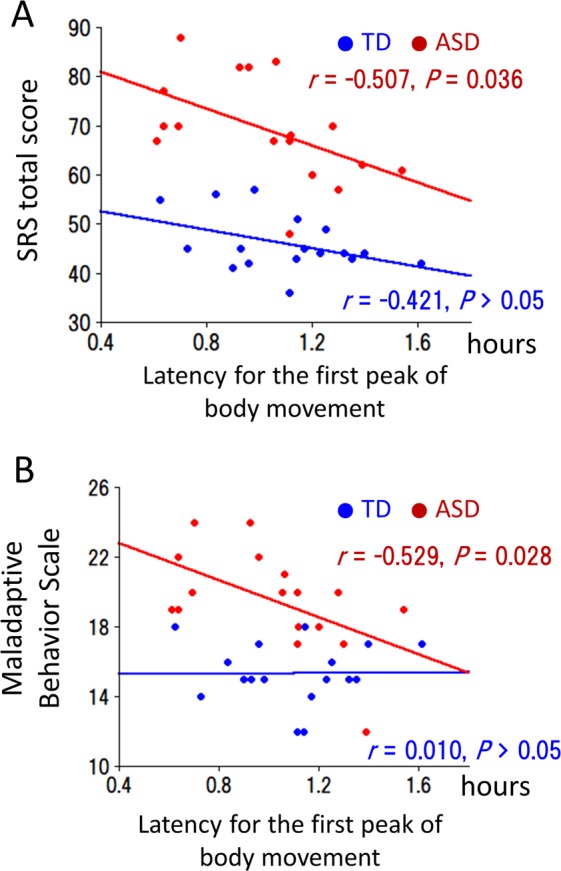
Relationship between the latency to the first peak of body movement and the severity of symptoms. (**A**) Relationship between the latency to the first peak of body movement and the SRS total score (a higher value indicated a lower social ability). (**B**) Relationship between the latency to the first peak of body movement and the MBS (a higher value indicated more severe maladaptive behaviour).

A significant negative correlation was observed between the latency to the first MI peak and the total SRS score in the children with ASD (*r* = −0.507, *P* = 0.036) but not in the TD children (*r* = −0.421, *P* > 0.05) (Fig. 4A). Therefore, the shorter latency to the first peak was associated with a lower social ability in children with ASD. Regarding the second peak, significant correlations were not observed in the children with ASD (*r* = −0.244, *P* > 0.05) or the TD children (*r* = −0.002, *P* > 0.05).

### Correlations between the latency to the peak of the movement index (MI) and maladaptive behaviour

Significant negative correlations were observed between the first MI latency peak and the MBS scores in the children with ASD (*r* = −0.529, *P* = 0.028) but not in the TD children (*r* = 0.010, *P* > 0.05) (Fig. 4B). Therefore, the shorter latency to the first peak was associated with maladaptive behaviour in children with ASD. Regarding the second peak, significant correlations were not observed in the children with ASD (*r* = −0.201, *P* > 0.05) or the TD children (*r* = 0.021, *P* > 0.05).

### The distribution of the movement index (MI) in TD children and children with ASD

**Figure 5:**
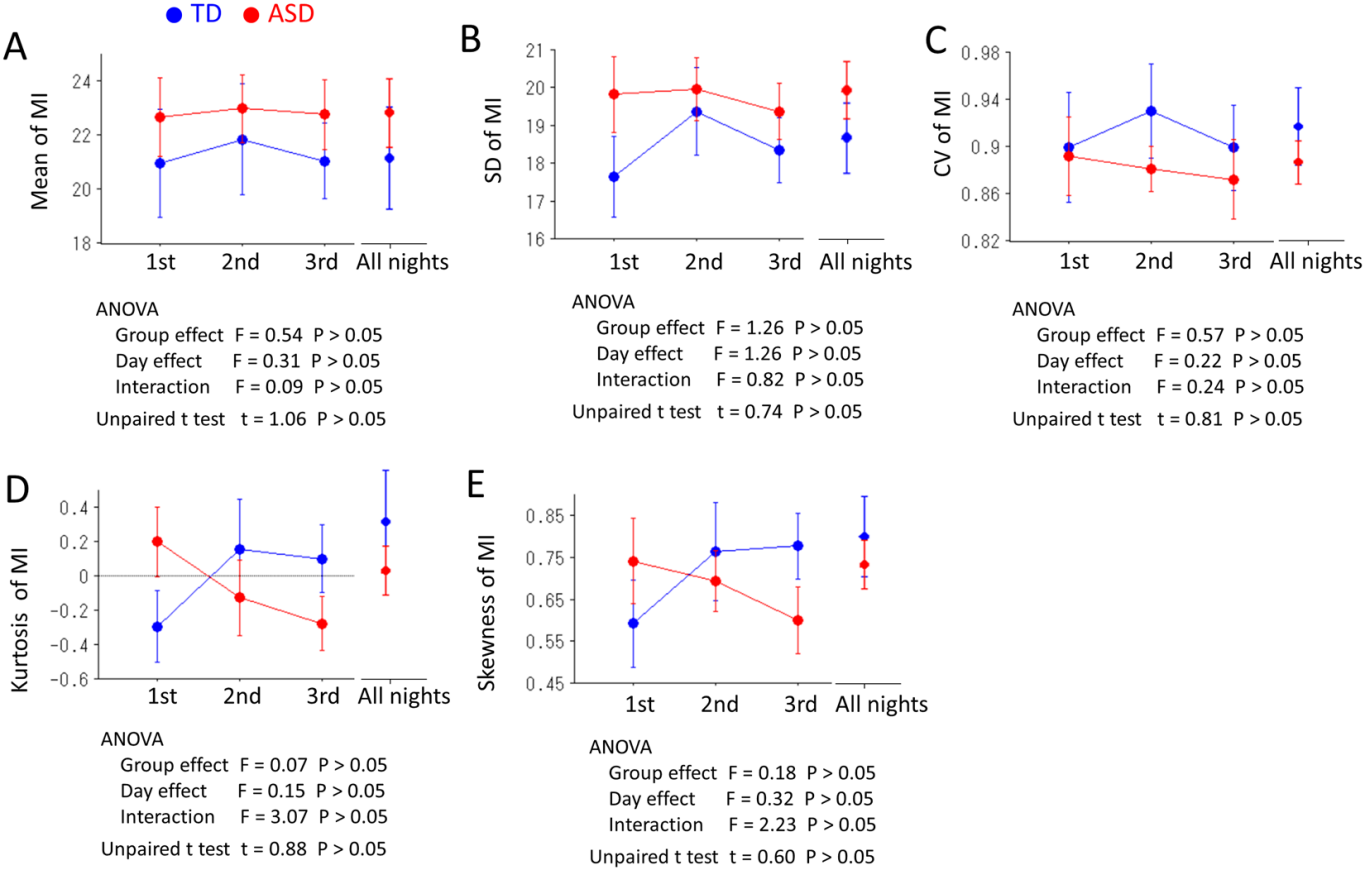
Two-way ANOVAs (three nights × two groups) revealed neither a significant main effect nor an interaction between the two factors for any indices of the distribution of the MI, i.e., (**A**) mean value, (**B**) SD, standard deviation, (**C**) CV, coefficient of variation, (**D**) kurtosis and (**E**) skewness for each night. Unpaired t-test between TD children and children with ASD for the combined data from all 3 nights also failed to demonstrate significant differences for any indices on the distribution of MI.

Two-way ANOVAs (three nights × two groups) revealed neither a significant main effect (*P* > 0.05) nor an interaction between the two factors (*P* > 0.05) for any indices on the distribution of MI (i.e., mean values, standard deviation (SD), coefficient of variation (CV), kurtosis and skewness of MI for each night (Fig. 5). The unpaired t-test between TD children and children with ASD for the combined data from all 3 nights also failed to demonstrate significant differences for any indices on the distribution of MI (Fig. 5). The Pearson correlation coefficients revealed no significant correlations (*P* > 0.05) between the indices on the distribution of MI and the behavioural indices (i.e., SRS, MBS and the attention-deficit hyperactivity disorder rating scale (ADHD-RS)) in the TD children and children with ASD (Table 2).

**Table 2:** Pearson correlation coefficients between the indices of the distribution of the MI and the behavioural indices.

	SRS total score	Vineland-II, MBS	ADHD-RS, total score
**TD (n = 17)**			
Mean of MI	−0.04	−0.03	0.11
SD of MI	−0.11	−0.10	−0.18
CV of MI	−0.08	−0.05	−0.18
Kurtosis	−0.17	0.05	−0.23
Skewness	−0.12	0.02	−0.17
**ASD (n = 17)**			
Mean of MI	0.35	0.16	−0.04
SD of MI	0.15	−0.04	−0.14
CV of MI	−0.45	−0.36	−0.16
Kurtosis	0.04	0.06	−0.04
Skewness	−0.15	−0.06	−0.07

There was no significant correlation (*P* > 0.05).

## Discussion

The present study is the first to report that a higher rate of body movement 2 to 3 hours after the first onset of body stillness was more prominent in children with ASD than in TD children. Even in children whose carers reported no apparent problems with daily sleep, the time course of body movements during night in young children with ASD differed from that in the TD children. In addition, the higher rate of body movement 0.5 to 1 hour after the first onset of body stillness was associated with a lower social ability and more frequent maladaptive behaviour in young children with ASD.

In general, both adults and children move through repeating cycles of deep and light sleep. Normal sleep involves cycles of light sleep (e.g., REM, sleep stage 1) and deep sleep (e.g., slow wave sleep). Cycles of deep and light sleep last for 30–50 minutes in babies and gradually increase in length throughout childhood^27^. Given that body movements are less frequent during deep sleep and more frequent during light sleep^22^, the repeating patterns of decreasing and increasing body movements demonstrated in the present study reflect the repeating cycles of deep and light sleep in young children. As children age, REM latency increases^27^ (i.e., shorter cycles mean immature sleep in young children); therefore, shorter first, second, third and fourth peaks of body movement may reflect a more immature sleep-related brain system. Short REM latencies have been reported in previous studies using PSG in children with ASD^7,28^. However, in the present study, we used only an accelerometer and could not accurately evaluate the REM latency. Nonetheless, our results demonstrated that the latency to the first peak of body movement tended to be shorter in children with ASD than in TD children, suggesting that REM latency tends to be shorter in children with ASD than in TD children and suggesting an immature sleep-related brain system in children with ASD. In addition, our results suggested that the severity of social symptoms in young children with ASD were associated with a shorter latency to the first nocturnal peak of body movement. These results suggest that a common pathophysiology might play an important role in both the abnormal distribution of motor activity nocturnal peaks and lower social skills in children with ASD. Atypical proprioceptive feedback in motor learning is thought to be associated with social impairment in ASD^29^. Therefore, atypical proprioceptive feedback might influence movements such as turning over in bed and may also influence the development of sociality in young children with ASD. This view is consistent with the results reported in previous studies of head micro-movements during arousal conditions in subjects with ASD^30^.

Regarding the time series analysis of body movements (Figs 2 and 3), in both the ASD and TD groups, higher peaks of body movements at 0.5 to 1 hour after the first onset of body stillness were associated with lower social function measured by the SRS and Vineland II. In both the ASD and TD groups, earlier onset of the first peaks of body movement might reflect the immaturity of the socially related brain in children. A possible reason why the difference was the most significant between the ASD and TD groups 2 to 3 hours after the first onset of body stillness is that the stably shorter period between the first and second peaks of body movement promoted higher peaks of body movements in these time windows in children with ASD. These higher peaks were not associated with symptom severity in children with ASD and therefore seem to be an ASD trait marker that cannot be measured using the SRS score. Unexpectedly, lower body movement 4 to 5 hours after the onset of first body stillness in bed was associated with a higher severity of social symptoms, as evaluated by the SRS and Vineland-II in children with ASD. During the latter half of sleep, slight differences in the sleep cycle become large differences (e.g., one cycle) due to the repetition of the cycle. Therefore, it is difficult to conclude how this result in this narrow time window reflects the differences in sleep phases. Alternatively, it may be possible that this result happened by chance in this narrow time window due to the multiple statistical analyses. Further study with a larger sample will be necessary to determine whether this unexpected result is replicable.

During the latter half of night, the ASD and TD children did not differ in the number of body movements. However, our results showed that a higher rate of body movement in the latter half of night was associated with a higher severity of social symptoms of ASD, suggesting that severe ASD symptoms are associated with a higher rate of the awake state, stage 1 sleep and REM sleep during the latter half of sleep^22^. Sleep fragmentation is suggested to disturb long-term hippocampus-dependent memory consolidation^31^. Intriguingly, a human study demonstrated that sleep enhances body movement memory obtained through the observation of movements in the absence of practice^32^. Therefore, it is tempting to hypothesize that lower social skills in children with ASD might partially be consequences of fragmented sleep preventing them from learning social body movement memory from early childhood.

With regard to head movement in infants, recent studies examined movement signatures of infants at high or low familial risk for autism (HR or LR) as they were listening to native language speech during a functional magnetic resonance imaging (MRI) scan, compared to simply sleeping during the resting-state fMRI scan^30,33^. In these studies, the quantitative characteristics of the subtle fluctuations of head movements were examined using the gamma probability distribution. Intriguingly, noisier movements were detected in HR infants (as early as 1–2 months) compared to LR infants, and these features could predict delays in early learning developmental trajectories^33^. In the present study, we also investigated the distribution signature of the MI using five indices related to the distribution of MI: mean values, standard deviation (SD), coefficients of variation (CV), kurtosis and skewness of the MI for each night and for the combined data from all 3 nights. However, we failed to demonstrate significant differences between TD children and children with ASD. A longer time window (9 hours) to calculate these indices of the distribution and a higher age range (5 to 8 years) in the present study might contribute to the inconsistent results.

The present study has some general limitations. First, using an accelerometer, body motion can be evaluated during sleep. However, simultaneous measurements with conventional PSG, which is necessary to evaluate the sleep structure, are lacking. Therefore, regarding the sleep structure, considerable caution should be exercised in drawing any definite conclusions about this structure based on our results from a waist-placed accelerometer in comparison with previous results from wrist-placed accelerometer studies. Second, some previous studies have reported prolonged sleep onset in ASD subjects^13,28^. However, we did not evaluate sleep onset latency in the present study because, in young children, defining the time at which the children go to bed to sleep is difficult because of their diverse sleeping habits. In addition, we did not ask about potential body rocking, sleep apnoea or restless legs syndrome, which might have influenced the study results. Further investigations using carer sleep diaries for children (e.g., When did the children go to their bedroom to sleep? When did they stop reading a book in bed? Are their moving their legs actions to seek relief from their discomfort?) and reliable sleep questionnaires (e.g., the Japanese Sleep Questionnaire for Preschoolers)^34^ with PSG are necessary. Third, most children with ASD examined in the present study were high functioning. Thus, the findings of this study may not apply to children with “Kanner’s autism”. In addition, we did not evaluate motor function such as motor assessment scales; therefore, further research that takes motor function into consideration is necessary. Fourth, we did not evaluate how many nights of usable recordings are required to obtain reliable body movements during sleep in young children. A previous study reported that five or more nights of usable recordings are required to estimate sleep patterns with activity monitoring in children and adolescents^35^. Therefore, data from five or more nights may be more reliable in this kind of study. Fifth, the amount of activity during the day (e.g., nap) was not objectively measured because we removed the accelerometer during the daytime. Daytime activity might affect body movement during sleep.

In conclusion, our results demonstrated a higher rate of body movement 2 to 3 hours after the first onset of body stillness in children with ASD compared to that in TD children. In addition, the higher rate of body movement 0.5 to 1 hour after the first onset of body stillness was associated with a lower social ability and lower adaptive behaviour in children with ASD. Although we could not draw any definite conclusions for sleep structure from our waist-placed accelerometer without validation using PSG, atypical nocturnal body movement could be an ASD state and trait marker in young children with ASD.

## Materials and Methods

### Participants

In total, 17 TD children with no reported behavioural or language problems participated in this cross-sectional study. The clinical group included 17 children with ASD who were recruited from Kanazawa University and prefectural hospitals in the Kanazawa or Toyama area. The ASD diagnosis was made according to the Diagnostic and Statistical Manual of Mental Disorders (5^th^ edition) (DSM-5) (the American Psychiatric Association, 2013), the Japanese translation of the Diagnostic Interview for Social and Communication Disorders (DISCO)^36^, or the Japanese translation of the Autism Diagnostic Observation Schedule–Generic (ADOS-G)^37^ (Kanekoshobo Co. Ltd.) and was conducted by a psychiatrist and a clinical speech therapist. All children fulfilled the diagnosis of childhood autism or atypical autism with DISCO. Only one subject did not meet the ADOS criteria for the autism or autism spectrum; however, he fulfilled the diagnosis of atypical autism with DISCO and autism spectrum disorder with DSM-5. Therefore, we included this subject as a child with ASD. Children who had psychiatric diseases other than ASD and ADHD were excluded using Mini International Neuropsychiatric Interview for Children and Adolescents^38^. Considering the frequent co-occurrence of ASD and ADHD symptoms, we did not exclude ASD patients with ADHD symptoms. Regarding the children’s general health status, any diseases currently under treatment were reported by the parents, and patients with diseases currently under treatment were excluded. Assessments were conducted by a psychiatrist and a clinical speech therapist. The children’s cognitive skills were assessed by the Japanese adaptation of the Kaufman Assessment Battery for Children (K-ABC)^39^. The typical sleep time of the participants was estimated based on carers’ reports. This typical sleep time is not the total time spent asleep (total sleep time) but the total amount of time spent in bed in recent weeks, which was reported by participants/carers. In addition, the carers were asked “Do you think the sleep of your child has been good recently?” and were required to rate their child’s sleep quality subjectively using a six-point rating scale (i.e., 1 = very bad, 2 = bad, 3 = somewhat bad, 4 = somewhat good, 5 = good and 6 = very good). Children in the TD and ASD groups were excluded from the study if a review of their medical history showed a history of epilepsy or intellectual disability or if they were taking psychotropic medications. Quantitative autistic traits in the children were assessed by the parents using the Japanese version^23^ of the SRS^23,24^. Higher scores on the SRS indicated a higher degree of social impairment. The raw scores of the SRS were converted to *T*-scores (with a mean of 50 and a standard deviation of 10) for each sex. The MBS from the Vineland-II was used to measure maladaptive behaviour^25^. Quantitative ADHD symptoms in the children were assessed using the Japanese version of the ADHD-RS^26^. Higher total scores on the ADHD-RS indicated a higher degree of ADHD symptoms.

The parents agreed to their child’s participation in the study with full knowledge of the experimental nature of the research. Written informed consent was obtained prior to participation in the study. The Ethics Committee of Kanazawa University Hospital approved the methods and procedures, which were performed in accordance with the Declaration of Helsinki. The demographic data of these subjects are presented in Table 1. The children’s sleep quality, which was rated by their carers, ranged from 4 to 6 (i.e., 4 = somewhat good, 5 = good, and 6 = very good). Therefore, no carers reported any problems with daily sleep. No carers reported that their children had obvious parasomnia symptoms, such as sleepwalking or night terrors, during our research. The number of preschool and school-aged children was 7 and 10 in TD children and 10 and 7 in children with ASD, respectively, and there was no difference between the 2 groups. Participants were usually able to participate in the school system (i.e., kindergarten or primary school).

### Motility levels during night

For the body movement measurements during night, we used a wristwatch-like accelerometer (Fig. 1A) attached to the waist. The device used in the present study was a Gen-2 GSR Wristband (Interuniversity Microelectronics Centre, Leuven, Kingdom of Belgium); the acceleration resolution was set at 0.016 G/s, and the time resolution was set at 32 Hz. Surface temperature was also recorded with the thermometer installed in the device. Just before the child got into bed, the parent attached a wearable device to the child. When the child got out of bed, the parent removed the wearable device. The time when the child entered and exited the bed was determined according to the temperature change. When the child had a sickness such as a common cold, their parents reported it, and we excluded the period from the analysis. The parents were asked to attach the device to their child’s waist using an adjustable shirt for at least 3 nights. The number of weekend days included in the three days for movement analysis was either 0, 1 or 2 days; the corresponding numbers of subjects were 12, 4 and 1 of the TD children and 8, 5 and 4 of the children with ASD, respectively, and there was no significant difference between the two groups. Although accelerometers are well tolerated in most children, some young children with ASD could not tolerate the device when placed on the wrist. One previous pilot study suggested that shoulder placement for actigraphy could be a reliable and alternative method for young children with ASD^40^. Another study reported that waist placement for actigraphy was successful for 7 consecutive days, even in 1.5-year-old TD children^41^. In the present study, although there was no validation using a waist-placed accelerometer to collect actigraphy data, we chose a waist placement for the accelerometer because it was more comfortable with the larger size of the device (Fig. 1A).

We analysed body movements after the first onset of body stillness for 9 hours and averaged this over 3 days (Fig. 1B). Because of the younger age range of the participants (5 to 8 years old), various rituals occurred before sleep onset (e.g., carer reading a book to the child), which could have confounded the determination of sleep onset latency. Therefore, in the present study, we disregarded the transition period from full wakefulness to sleep; we defined the first onset of body stillness in the bed as the baseline time (i.e., time 0) and evaluated the time series of the body movements.

The off-line analysis of the time-series data from the 3D accelerometer was performed with Brain Vision Analyzer (Brain Products GmbH, Gilching, Germany) and MATLAB (MathWorks, Natick MA). The first onset of body stillness in the bed was defined as the time when the accelerometer reported no obvious continuous body movements for ten minutes. Nine hours of time-series data were recorded after the first onset of body stillness for further analysis. First, the time-series data recorded by the 3D accelerometers at a sampling rate of 32 Hz were reduced to 1 Hz by block averaging each 1 sec period for each dimensional accelerometer. Second, to exclude the effect of sustained gravitational acceleration, low frequencies (0.0028 Hz) were filtered from each dimensional accelerometer to emphasize the changes in acceleration. Third, the root mean square (RMS) value of the data recorded by the 3D accelerometers was calculated for each time point. Fourth, for these RMS values, we defined the threshold (0.1 G/s). Specifically, periods over 0.1 G/s were considered periods of body movement. Fifth, the ratio of the body movement period in 20 minutes was calculated continuously for 9 hours using the sliding window method (i.e., sliding with a 1 sec time resolution). We named this ratio the “movement index (MI)”. The averaged time-series data (9-hour period after the first onset of body stillness) of the movement index (MI) averaged over three nights were used for further analysis (Fig. 1B).

### Statistical analysis

As shown in Fig. 1B, periodic increases in body movements that might correspond to REM sleep periods were observed in each subject. The MI of the TD children and children with ASD were compared with unpaired two-tailed *t*-tests for each time window during the 9-hour period after the first onset of body stillness. To investigate the relationship between the MI and the behavioural indices (SRS, MBS and ADHD-RS), we calculated the Pearson correlation coefficient between the MI and each of the indices for each time window during the 9-hour period after the first onset of body stillness in the TD children and children with ASD. In this exploratory analysis, *P* < 0.05 was considered to indicate statistical significance. In a complementary analysis, we identified the latency to the first peak of the MI from 0.5 to 2.0 hours after the first onset of body stillness, and the second peak occurred from 1.5 to 3.5 hours after the first onset of body stillness. These latencies were compared between the two groups using unpaired two-tailed *t*-tests. To investigate the relationship between the MI and the behavioural indices (SRS subscores and MBS), we calculated the Pearson correlation coefficients between the latency to the first and second peaks of the MI and the behavioural indices in the TD children and children with ASD. In this complementary analysis, *P* < 0.05 was considered to indicate statistical significance.

To test the differences in the distribution of the MI during night between the two groups, two-way ANOVAs for each night and an unpaired t-test for the combined data from all 3 nights were performed (three nights × two groups) for indices of the distribution of MI, i.e., mean value, standard deviation (SD), coefficient of variation (CV), kurtosis and skewness of the MI for each night. The within-subject factor was the three nights, whereas the between-subject factor was the group effect (TD vs. ASD). The significance level was set at 0.05. To investigate the relationship between the mean values (for three nights) of indices about the distribution of the MI and the behavioural indices (SRS, MBS and ADHD-RS), we calculated the Pearson correlation coefficients between them in the TD children and children with ASD. In this exploratory analysis, *P* < 0.05 was considered to indicate statistical significance.

## Supplementary information

Supplementary FigureS1

## Data Availability

The datasets used and analysed in the current study are available from the corresponding author upon reasonable request.
