# Supplementary material for: Atypical body movements during night in young children with autism spectrum disorder: a pilot study
Source: Sci Rep. 2019 May 6;9:6999. doi: 10.1038/s41598-019-43397-y (PMC6502823; doi:10.1038/s41598-019-43397-y)
Supplement: Supplementary file 1 — Supplementary FigureS1 [file 41598_2019_43397_MOESM1_ESM.docx]

**Supplementary information**

**Title: Atypical body movements during night in young children** **with autism spectrum disorder: a pilot study**

Running title: Atypical body movement during night in autism

Nobushige Naito^1^, Mitsuru Kikuchi^1,2^*, Yuko Yoshimura^3^, Hirokazu Kumazaki^2^, Sachiko Kitagawa^2^, Takashi Ikeda^2^, Chiaki Hasegawa^2^, Daisuke N. Saito^2^, Sarah Tomiyama^2^, Yoshio Minabe^1,2^

1) Department of Psychiatry & Behavioral Science, Graduate School of Medical Science, Kanazawa University, Kanazawa, 920-8640, Japan

2) Research Center for Child Mental Development, Kanazawa University, Kanazawa, 920-8640, Japan

3) Institute of Human and Social Sciences, Kanazawa University, Kanazawa, 920-1192, Japan

* Corresponding author:

Mitsuru Kikuchi

Research Center for Child Mental Development, Kanazawa

University, 13-1 Takara-machi, Kanazawa 920-8640, Japan

E-mail address: mitsuruk@med.kanazawa-u.ac.jp

Tel.: +81-76-265-2856; Fax: +81-76-234-4213

Supplementary figure


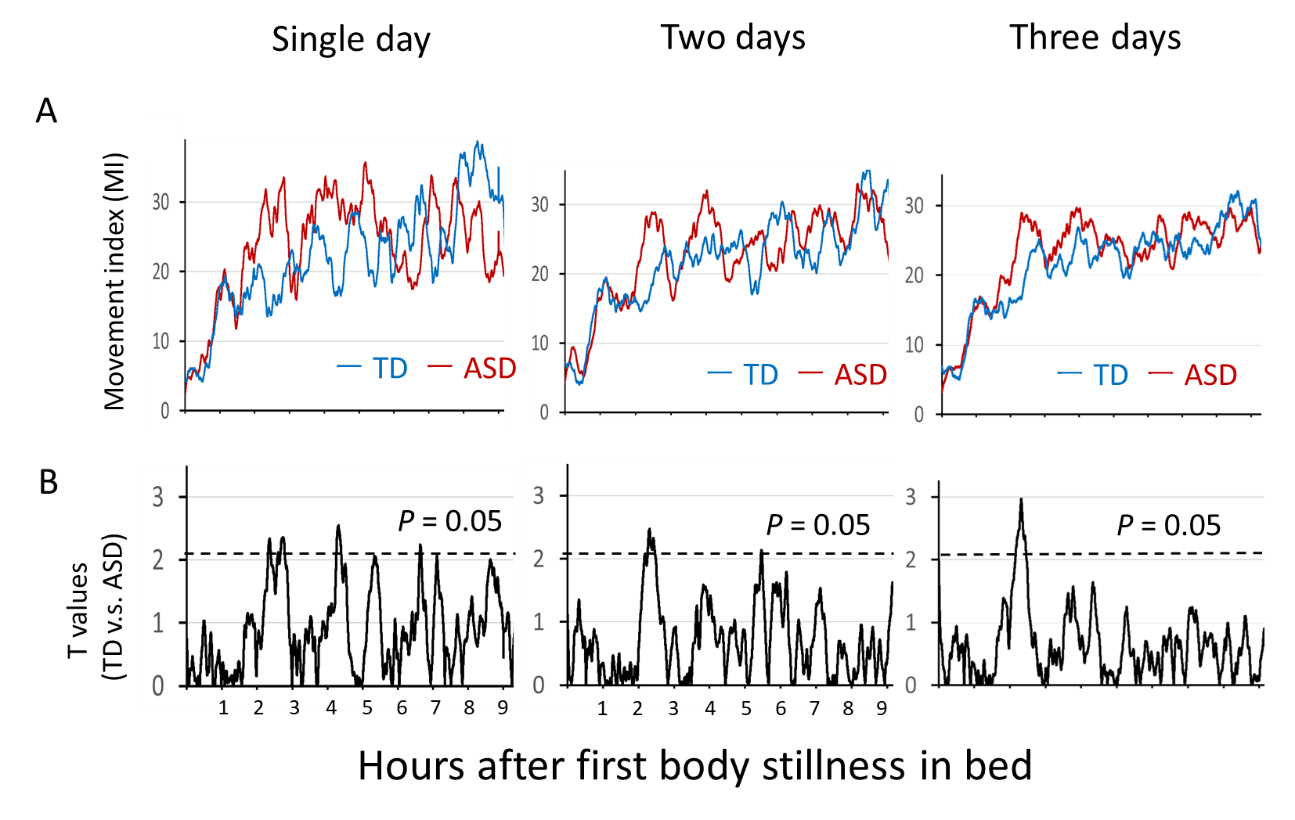


**Figure S1**. Overall averaged time-series data of the movement index (MI) after the first onset of body stillness in TD children (n = 17) and children with ASD (n = 17) using single day data (left), two days’ data (middle) and three days’ data (right). (A) The blue line shows the time series of the MI in the TD children, and the red line shows the time series of the MI in the children with ASD. (B) Time-series data of the MI in the TD children and children with ASD were compared using unpaired two-tailed t-tests for each time window (20 minutes). Broken lines show the threshold of P = 0.05 (t = 2.04). As the number of days increased, the significant difference after 2-3 hours between TD children and children with ASD became clearer and the other spiky T value waveform decreased.
